# Linking emotions to behaviors through deep transfer learning

**DOI:** 10.7717/peerj-cs.246

**Published:** 2020-01-06

**Authors:** Haoqi Li, Brian Baucom, Panayiotis Georgiou

**Affiliations:** 1 Department of Electrical and Computer Engineering, University of Southern California, Los Angeles, CA, United States of America; 2 Department of Psychology, University of Utah, Salt Lake City, UT, United States of America

**Keywords:** Behavior quantification, Emotion, Affective computing, Neural networks, Couples therapy

## Abstract

Human behavior refers to the way humans act and interact. Understanding human behavior is a cornerstone of observational practice, especially in psychotherapy. An important cue of behavior analysis is the dynamical changes of emotions during the conversation. Domain experts integrate emotional information in a highly nonlinear manner; thus, it is challenging to explicitly quantify the relationship between emotions and behaviors. In this work, we employ deep transfer learning to analyze their inferential capacity and contextual importance. We first train a network to quantify emotions from acoustic signals and then use information from the emotion recognition network as features for behavior recognition. We treat this emotion-related information as behavioral primitives and further train higher level layers towards behavior quantification. Through our analysis, we find that emotion-related information is an important cue for behavior recognition. Further, we investigate the importance of emotional-context in the expression of behavior by constraining (or not) the neural networks’ contextual view of the data. This demonstrates that the sequence of emotions is critical in behavior expression. To achieve these frameworks we employ hybrid architectures of convolutional networks and recurrent networks to extract emotion-related behavior primitives and facilitate automatic behavior recognition from speech.

## Introduction

Human communication includes a range of cues from lexical, acoustic and prosodic, turn taking and emotions to complex behaviors. Behaviors encode many domain-specific aspects of the internal user state, from highly complex interaction dynamics to expressed emotions. These are encoded at multiple resolutions, time scales, and with different levels of complexity. For example, a short speech signal or a single uttered word can convey basic emotions (Ekman, 1992a; Ekman, 1992b). More complex behaviors require domain specific knowledge and longer observation windows for recognition. This is especially true in task specific behaviors of interest in observational treatment for psychotherapy such as in couples’ therapy (Christensen et al., 2004) and suicide risk assessment (Cummins et al., 2015). Behaviors encompass a rich set of information that includes the dynamics of interlocutors and their emotional states, and can often be domain specific. The evaluation and identification of domain specific behaviors (e.g., blame, suicide ideation) can facilitate effective and specific treatments by psychologists. During the observational treatment, annotation of human behavior is a time consuming and complex task. Thus, there have been efforts on automatically recognizing human emotion and behavior states, which resulted in vibrant research topics such as affective computing (Tao & Tan, 2005; Picard, 2003; Sander & Scherer, 2014), social signal processing (Vinciarelli, Pantic & Bourlard, 2009), and behavioral signal processing (BSP) (Narayanan & Georgiou, 2013; Georgiou, Black & Narayanan, 2011). In the task of speech emotion recognition (SER), researchers are combining machine learning techniques to build reliable and accurate affect recognition systems (Schuller, 2018). In the BSP domain, through domain-specific focus on areas such as human communication, mental health and psychology, research targets advances of understanding of higher complexity constructs and helps psychologists to observe and evaluate domain-specific behaviors.

However, despite these efforts on automatic emotion and behavior recognition (see ‘Related Work’), there has been less work on examining the relationship between these two. In fact, many domain specific annotation manuals and instruments (Heavey, Gill & Christensen, 2002; Jones & Christensen, 1998; Heyman, 2004) have clear descriptions that state specific basic emotions can be indicators of certain behaviors. Such descriptions are also congruent with how humans process information. For example, when domain experts attempt to quantify complex behaviors, they often employ affective information within the context of the interaction at varying timescales to estimate behaviors of interest (Narayanan & Georgiou, 2013; Tseng et al., 2016).

Moreover, the relationship between behavior and emotion provides an opportunity for (i) transfer learning by employing emotion data, that is easier to obtain, annotate, and less subjective, as the initial modeling task; and (ii) employing emotional information as building blocks, or primitive features, that can describe behavior.

The purpose of this work is to explore the relationship between emotion and behavior through deep neural networks, and further the employ emotion-related information towards behavior quantification. There are many notions of what an “emotion” is. For the purpose of this paper and most research in the field (El Ayadi, Kamel & Karray, 2011; Schuller, 2018), the focus is on *basic emotions*, which are defined as cross-culturally recognizable. One commonly used discrete categorization is by Ekman (1992a); Ekman (1992b), in which six basic emotions are identified as anger, disgust, fear, happiness, sadness, and surprise. According to theories (Schacter, Gilbert & Wegner, 2011; Scherer, 2005), emotions are states of feeling that result in physical and psychological changes that influence our behaviors.

Behavior, on the other hand, encodes many more layers of complexity: the dynamics of the interlocutors, their perception, appraisal, and expression of emotion, their thinking and problem-solving intents, skills and creativity, the context and knowledge of interlocutors, and their abilities towards emotion regulation (Baumeister et al., 2007; Baumeister et al., 2010). Behaviors are also domain dependent. In addiction (Baer et al., 2009), for example, a therapist will mostly be interested in the language which reflects changes of addictive habits. In suicide prevention (Cummins et al., 2015), reasons for living and emotional bond are more relevant. In doctor-patient interactions, empathy or bedside manners are more applicable.

In this paper, we will first address the task of basic emotion recognition from speech. Thus we will discuss literature on the notion of emotion (see ‘Emotions’) and prior work on emotion recognition (see ‘Emotion quantification from speech’). We will then, as our first scientific contribution, describe a system that can label emotional speech (see ‘Emotion recognition’).

The focus of this paper, however, is to address the more complex task of behavior analysis. Given behavior is very related to the dynamics, perception, and expression of emotions (Schacter, Gilbert & Wegner, 2011), we believe a study is overdue in establishing the degree to which emotions can predict behavior. We will therefore introduce more analytically the notion of behavior (see ‘Behaviour’) and describe prior work in behavior recognition (see ‘Behavior quantification from speech’), mainly from speech signals. The second task of this paper will be in establishing a model that can predict behaviors from basic emotions. We will investigate the emotion-to-behavior aspects in two ways: we will first assume that the discrete emotional labels directly affect behavior (see ‘Context-dependent behavior recognition from emotion labels’). We will further investigate if an embedding from the emotion system, representing behaviors but encompassing a wider range of information, can better encode behaviorally meaningful information (see ‘Context-dependent behavior recognition from emotion-embeddings’).

In addition, the notion that behavior is highly dependent on emotional expression also raises the question of how important the sequence of emotional content is in defining behavior. We will investigate this through progressively removing the context from the sequence of emotions in the emotion-to-behavior system (see ‘Reduced context-dependent behavior recognition from emotion-informed embeddings’) and study how this affects the automatic behavior classification performance.

## Background

### Emotions

There is no consensus in the literature on a specific definition of emotion. An “emotion” is often taken for granted in itself and, most often, is defined with reference to a list of descriptors such as anger, disgust, happiness, and sadness etc. (Cabanac, 2002). Oatley & Jenkins (1996) distinguish emotion from mood or preference by the duration of each kind of state. Two emotion representation models are commonly employed in practice (Schuller, 2018). One is based on the discrete emotion theory, where six basic emotions are isolated from each other, and researchers assume that any emotion can be represented as a mixture of the basic emotions (Cowie et al., 2001). The other model defines emotions via continuous values corresponding to different dimensions which assumes emotions change in a continuous manner and have strong internal connections but blurred boundaries between each other. The two most common dimensions are arousal and valence (Schlosberg, 1954).

In our work, following related literature, we will refer to basic emotions as emotions that are expressed and perceived through a short observation window. Annotations of such emotions take place without context to ensure that time-scales, back-and-forth interaction dynamics, and domain-specificity is not captured.

### Behavior

Behavior is the output of information and signals including but not limited to those: (i) manifested in both overt and covert multimodal cues (“expressions”); and (ii) processed and used by humans explicitly or implicitly (“experience” and “judgment”) (Narayanan & Georgiou, 2013; Baumeister et al., 2010). Behaviors encompass significant degrees of emotional perception, facilitation, thinking, understanding and regulation, and are functions of dynamic interactions (Baumeister et al., 2007). Further, such complex behaviors are increasingly domain specific and subjective.

### Link between emotions and behavior

Emotions can change frequently and quickly in a short time period (Ekman, 1992a; Mower & Narayanan, 2011). They are internal states that we perceive or express (e.g., through voice or gesture) but are not interactive and actionable. Behaviors, on the other hand, include highly complex dynamics, information from explicit and implicit aspects, are exhibited over longer time scales, and are highly domain specific.

For instance, “happiness”, as one of the emotional states, is brought about by generally positive feelings. While within couples therapy domain, behavior “positivity” is defined in (Heavey, Gill & Christensen, 2002; Jones & Christensen, 1998) as “Overtly expresses warmth, support, acceptance, affection, positive negotiation”.

**Figure 1:**
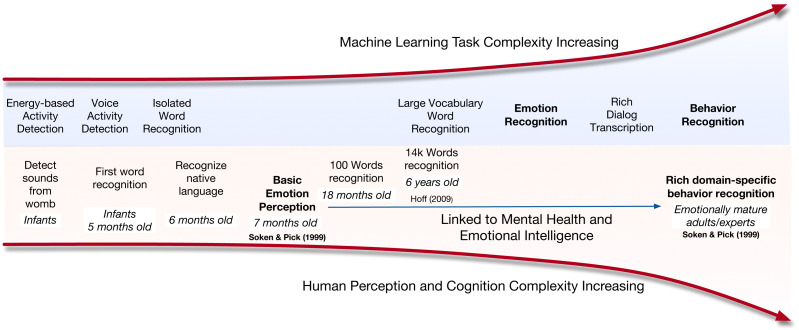
Illustration of task complexity or age of acquisition for machines and humans.

Those differences apply to both human cognition and machine learning aspects of speech capture, emotion recognition and behavior understanding as shown in Fig. 1 (Soken & Pick, 1999; Hoff, 2009). The increased complexity and contextualization of behavior can be seen both in humans as well as machines. For example, babies start to develop basic emotion perception at the age of seven months (Soken & Pick, 1999). However, it takes emotionally mature and emotionally intelligent humans and often trained domain experts to perceive domain-specific behaviors. In Fig. 1, we illustrate the complexity for machine processing along with the age-of-acquisition for humans. We see a parallel in the increase in demands of identifying behavior in both cases.

### Motivations and goals of this work

The relationship between emotion and behavior is usually implicit and highly nonlinear. Investigating explicit and quantitative associations between behavior and emotions is thus challenging.

In this work, based on the deep neural networks’ (DNNs) underlying representative capability (Bengio, Courville & Vincent, 2013; Bengio, 2012), we try to analyze and interpret the relationship between emotion and behavior information through data-driven methods. We investigate the possibility of using transfer learning by employing emotion data as emotional related building blocks, or primitive features, that can describe behavior. Further, we design a deep learning framework that employs a hybrid network structure containing context dependent and reduced contextualization causality models to quantitatively analyze the relationship between basic emotions and complex behaviors.

## Related Work

Researchers are combining machine learning techniques to build reliable and accurate emotion and behavior recognition systems. Speech emotion recognition (SER) systems, of importance in human-computer interactions, enable agents and dialogue systems to act in a more human-like manner as conversational partners (Schuller, 2018). On the other hand, in the domain of behavior signal processing (BSP), efforts have been made in quantitatively understanding and modeling typical, atypical, and distressed human behavior with a specific focus on verbal and non-verbal communicative, affective, and social behaviors (Narayanan & Georgiou, 2013). We will briefly review the related work in the following aspects.

### Emotion quantification from speech

A dominant modality for emotion expression is speech (Cowie & Cornelius, 2003). Significant efforts (El Ayadi, Kamel & Karray, 2011; Beale & Peter, 2008; Schuller et al., 2011) have focused on automatic speech emotion recognition. Traditional emotion recognition systems usually rely on a two-stage approach, in which the feature extraction and classifier training are conducted separately. Recently, deep learning has demonstrated promise in emotion classification tasks (Han, Yu & Tashev, 2014; Le & Provost, 2013). Convolutional neural networks (CNNs) have been shown to be particularly effective in learning affective representations directly from speech spectral features (Mao et al., 2014; Anand & Verma, 2015; Huang & Narayanan, 2017a; Zheng, Yu & Zou, 2015; Aldeneh & Provost, 2017). Mao et al. (2014) proposed to learn CNN filters on spectrally whitened spectrograms by an auto-encoder through unsupervised manners. Aldeneh & Provost (2017) showed that CNNs can be directly applied to temporal low-level acoustic features to identify emotionally salient regions. Anand & Verma (2015) and Huang & Narayanan (2017a) compared multiple kinds of convolutional kernel operations, and showed that the full-spectrum temporal convolution is more favorable for speech emotion recognition tasks. In addition, models with hidden Markov model (HMM) (Schuller, Rigoll & Lang, 2003), recurrent neural networks (RNNs) (Wöllmer et al., 2010; Metallinou et al., 2012; Lee & Tashev, 2015) and the hybrid neural network combining CNNs and RNNs (Lim, Jang & Lee, 2016; Huang & Narayanan, 2017b) have also been employed to model emotion affect.

### Behavior quantification from speech

Behavioral signal processing (BSP) (Narayanan & Georgiou, 2013; Georgiou, Black & Narayanan, 2011) can play a central role in informing human assessment and decision making, especially in assisting domain specialists to observe, evaluate and identify domain-specific human behaviors exhibited over longer time scales. For example, in couples therapy (Black et al., 2013; Nasir et al., 2017b), depression (Gupta et al., 2014; Nasir et al., 2016; Stasak et al., 2016; Tanaka, Yamamoto & Haruno, 2017) and suicide risk assessment (Cummins et al., 2015; Venek et al., 2017; Nasir et al., 2018; Nasir et al., 2017a), behavior analysis systems help psychologists observe and evaluate domain-specific behaviors during interactions. Li, Baucom & Georgiou (2016) proposed sparsely connected and disjointly trained deep neural networks to deal with the low-resource data issue in behavior understanding. Unsupervised (Li, Baucom & Georgiou, 2017) and out-of-domain transfer learning (Tseng, Baucom & Georgiou, 2018) have also been employed on behavior understanding tasks. Despite these important and encouraging steps towards behavior quantification, obstacles still remain. Due to the end-to-end nature of recent efforts, low-resource data becomes a dominant limitation (Li, Baucom & Georgiou, 2016; Collobert et al., 2011; Soltau, Liao & Sak, 2017; Heyman et al., 2001). This is exacerbated in BSP scenario by the difficulty of obtaining data due to privacy constraints (Lustgarten, 2015; Narayanan & Georgiou, 2013). Challenges with subjectivity and low interannotator agreement (Busso & Narayanan, 2008; Tseng et al., 2016), especially in micro and macro annotation complicate the learning task. Further, and importantly such end-to-end systems reduce interpretability generalizability and domain transfer (Sculley et al., 2015).

### Linking emotion and behavior quantification

As mentioned before, domain experts employ information within the context of the interaction at varying timescales to estimate the behaviors of interest (Narayanan & Georgiou, 2013; Tseng et al., 2016). Specific short-term affect, e.g., certain basic emotions, can be indicators of some complex long-term behaviors during manual annotation process (Heavey, Gill & Christensen, 2002; Jones & Christensen, 1998; Heyman, 2004). These vary according to the behavior; for example, negativity is often associated with localized cues (Carney, Colvin & Hall, 2007), demand and withdrawal require more context (Heavey, Christensen & Malamuth, 1995), and coercion requires a much longer context beyond a single interaction (Feinberg, Kan & Hetherington, 2007). Chakravarthula et al. (2019) analyzed behaviors, such as “anger” and “satisfaction”, and found that negative behaviors could be quantified using short observation length whereas positive and problem solving behaviors required much longer observation.

In addition, Baumeister et al. (2007) and Baumeister et al. (2010) discussed two kinds of theories: the direct causality model and inner feedback model. Both models emphasize the existence of a relationship between basic emotion and complex behavior. Literature from psychology (Dunlop, Wakefield & Kashima, 2008; Burum & Goldfried, 2007) and social science (Spector & Fox, 2002) also showed that emotion can have impacts and further shape certain complex human behaviors. To connect basic emotion with more complex affective states, Carrillo et al. (2016) identified a relationship between emotional intensity and mood through lexical modality. Khorram et al. (2018) verified the significant correlation between predicted emotion and mood state for individuals with bipolar disorder on acoustic modality. All these indicate that the aggregation and link between basic emotions and complex behaviors is of interest and should be examined.

## Proposed Work: Behavioral Primitives

Our work consists of three studies for estimation of behavior through emotion information as follows:

1. **Context-dependent behavior from emotion labels:** Basic emotion affect labels are directly used to predict long-term behavior labels through a recurrent neural network. This model is used to investigate whether the basic emotion states can be sufficient to infer behaviors.
2. **Context-dependent behavior from emotion-informed embeddings:** Instead of directly using the basic emotion affect labels, we utilize emotion-informed embeddings towards the prediction of behaviors.
3. **Reduced context-dependent behavior from emotion-informed embeddings:** Similar to (2) above, we employ emotion-informed embeddings. In this case, however, we investigate the importance of context, by progressively reducing the context provided to the neural network in predicting behavior.

For all three methods, we utilize a hybrid model of convolution and recurrent neural networks that we will describe in more detail below.

Through our work, both emotion labels and emotionally informed embeddings will be regarded as a type of behavior primitive, that we call ***Basic Affect Behavioral Primitive Information (or Behavioral Primitives for short, BP)***.

An important step in obtaining the above BP is the underlying emotion recognition system. We thus first propose and train a robust ***Multi-Emotion Regression Network (ER)*** using convolutional neural network (CNN), which is described in detail in the following subsection.

### Emotion recognition

In order to extract emotionally informed embeddings and labels, we propose a CNN based ***Multi-Emotion Regression Network (ER)***. The ER model has a similar architecture as (Aldeneh & Provost, 2017), except that we use one-dimensional (1D) CNN kernels and train the network through a regression task. The CNN kernel filter should include entire spectrum information per scan, and shift along the temporal axis, which performs better than other kernel structures according to Huang & Narayanan (2017a).

Our model has three components: (1) stacked 1D convolutional layers; (2) an adaptive max pooling layer; (3) stacked dense layers. The input acoustic features are first processed by multiple stacked 1D convolution layers. Filters with different weights are employed to extract different information from the same input sample. Then, one adaptive max pooling layer is employed to further propagate 1D CNN outputs with the largest value. This is further processed through dense layers to generate the emotional ratings at short-term segment level. The adaptive max pooling layer over time is one of the key components of this and all following models: first, it can cope with variable length signals and produce fixed size embeddings for subsequent dense layers; Second, it only returns the maximum feature within the sample to ensure only the more relevant emotionally salient information is propagated through training.

We train this model as one regression model which predicts the annotation ratings of all emotions jointly. Analogous to the continuous emotion representation model (Schlosberg, 1954), this multi-emotion joint training framework can utilize strong bonds but blurred boundaries within emotions to learn the embeddings. Through this joint training process, the model can integrate the relationship across different emotions, and hopefully obtain an affective-rich embedding.

In addition, to evaluate the performance of proposed ER , we also build multiple binary, single-emotion, classification models (***Single-Emotion Classification Network (EC)***). The EC model is modified based on pre-trained ER by replacing the last linear layer with new fully connected layers to classify each single emotion independently. During training, the back propagation only updates the newly added linear layers without changing the weights of pre-trained ER model. In this case, the loss from different emotions is not entangled and the weights will be optimized towards each emotion separately. More details of experiments and results comparison are described in ‘Experiments and Results Discussion’.

**Figure 2:**
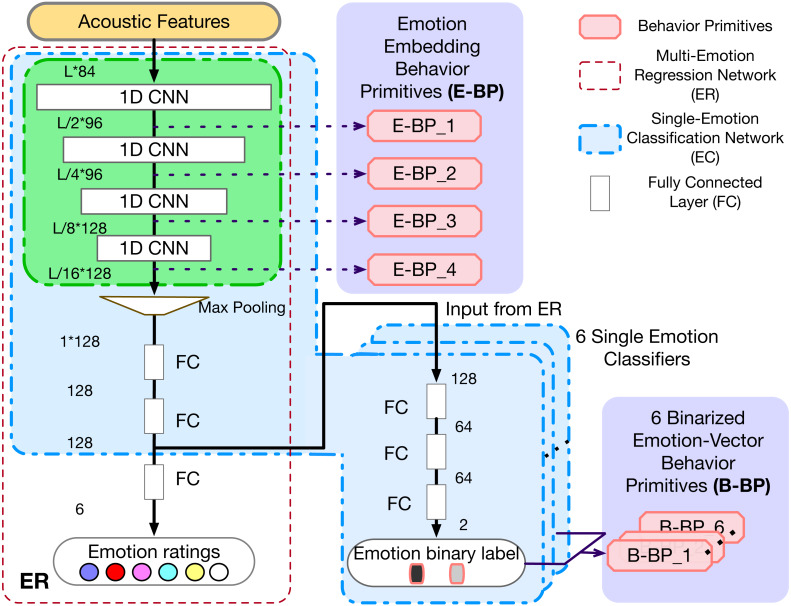
Models of ER, EC and two kinds of BPs. L is the input feature length.

As mentioned before, we employ *two kinds of behavioral primitives* in order to investigate the relationship between emotions and behaviors, and the selection of these two kinds of BP arises through the discrete, EC, and continuous, ER, emotion representation models. The two kinds of BP are: (1) The discrete vector representation of predicted emotion labels, denoted as B-BP_k, from the Single-Emotion Classification Network (EC), where *k* means *k*^th^ basic emotion; and (2) The output embeddings of the CNN layers, denoted as E-BP_ *l*, from the ***Multi-Emotion Regression Network system (ER)***, where *l* represents the output from *l*^th^ CNN layer. All these are illustrated in Fig. 2.

### Behavior recognition through emotion-based behavior primitives

We now describe three architectures for estimating behavior through ***Basic Affect Behavioral Primitive Information (or Behavioral Primitives for short, BP)***. The three methods employ full context of the emotion labels from the ***Single-Emotion Classification Network (EC)***, the full context from the embeddings of the ***Multi-Emotion Regression Network (ER)*** system, and increasingly reduced context from the ***Multi-Emotion Regression Network system (ER)***.

#### Context-dependent behavior recognition from emotion labels

In this approach, the binarized predicted labels from the EC system are employed to predict long-term behaviors via sequential models in order to investigate relationships between emotions and behaviors. Such a design can inform the degree to which short-term emotion can influence behaviors. It can also provide some interpretability of the employed information for decision making, over end-to-end systems that generate predictions directly from the audio features.

**Figure 3:**
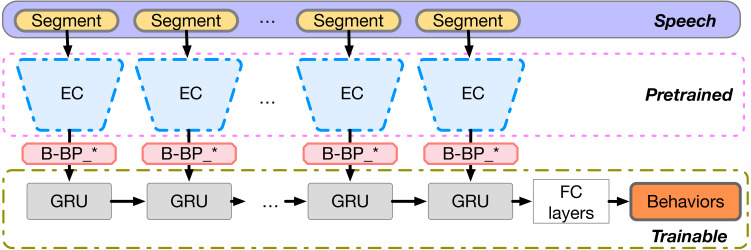
B-BP based context-dependent behavior recognition model.

We utilize the ***Single-Emotion Classification Network (EC)*** described in the previous section to obtain the predicted ***Binarized Emotion-Vector Behavior Primitives (B-BP)*** on shorter speech segment windows as behavioral primitives. These are extracted from the longer signals that describe the behavioral corpus and are utilized, preserving sequence, hence context, within a recurrent neural network for predicting the behavior labels. Figure 3 illustrates the network architecture and B-BP_* means the concatenation of all B-BP_k, where k ranges from 1 to 6.

In short, the B-BP vectors are fed into a stack of gated recurrent units (GRUs), followed by a densely connected layer which maps the last hidden state of the top recurrent layer to behavior label outputs. GRUs were introduced in Chung et al. (2014) as one attempt to alleviate the issue of vanishing gradient in standard vanilla recurrent neural networks and to reduce the number of parameters over long short-term memory (LSTM) neurons. GRUs have a linear shortcut through timesteps which avoids the decay and thus promotes gradient flow. In this model, only the sequential GRU components and subsequent dense layers are trainable, while the EC networks remain fixed.

#### Context-dependent behavior recognition from emotion-embeddings

It is widely understood that information closer to the output layer is more tied to the output labels while closer to the input layer information is less constrained and contains more information about the input signals. In our ER network, the closer we are to the output, the more raw information included in the signal is removed and the more we are constrained to the basic emotions. Given that we are not directly interested in the emotion labels, but in employing such relevant information for behavior, it makes sense to employ layers below the last output layer to capture more behavior-relevant information closer to its raw form. Thus, instead of using the binary values representing the absence or existence of the basic emotions, we can instead employ ***Emotion-Embedding Behavior Primitives (E-EBP)*** as the input representation.

**Figure 4:**
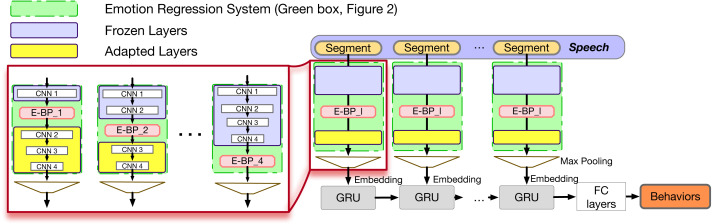
E-BP based context-dependent behavior recognition model. E-BP_ *l* is the output from *l*^th^ pretrained CNN layer. In practice multiple E-BP_ *l* can be employed at the same time through concatenation. In this work we only employ the output of a single layer at a time.

The structure of the system is illustrated in Fig. 4. After pretraining the ER , we keep some layers of that system fixed, and employ their embeddings as the Emotion-Embedding Behavior Primitives. We will discuss the number of fixed layers in the experiments section. This E-BP serves as the input of the subsequent, trainable, convolutional and recurrent networks.

The overall system is trained to predict the longer-term behavior states. By varying the number of layers that remain unchanged in the ER system and using different embeddings from different layers for the behavior recognition task we can identify the best embeddings under the same overall number of parameters and network architecture.

The motivation of the above is that the fixed ER encoding module is focusing on learning emotional affect information, which can be related but not directly linked with behaviors. By not using the final layer, we are employing a more raw form of the emotion-related information, without extreme information reduction, that allows for more flexibility in learning by the subsequent behavior recognition network. This allows for transfer learning (Torrey & Shavlik, 2010) from one domain (emotions) to another related domain (behaviors). Thus, this model investigates the possibility of using transfer learning by employing emotional information as “building blocks” to describe behavior.

#### Reduced context-dependent behavior recognition from emotion-informed embeddings

In the above work, we assume that the sequence of the behavior indicators (embeddings or emotions) is important. To verify the need for such an assumption, in this section, we propose varying the degree of employed context. Through quantification, we analyze the time-scales at which the amount of sequential context affects the estimation of the underlying behavioral states.

**Figure 5:**
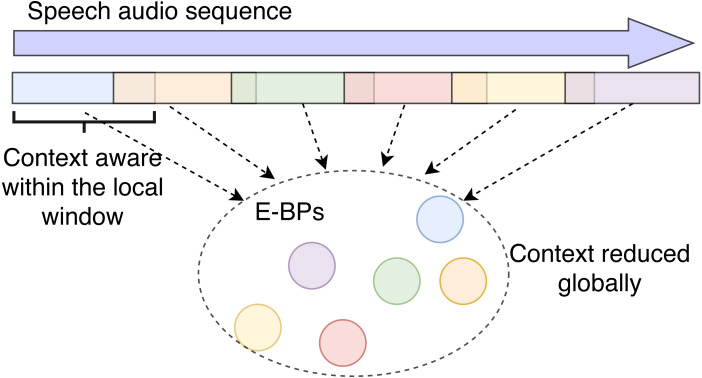
Illustration of local context awareness and global context reduction. In previous sections, the E-BPs (and B-BPs) are passed to a GRU that preserves their sequences. Here they are processed through pooling and context is removed.

In this proposed model, we design a network that can only preserve local context. The overall order of the embeddings extracted from the different local segments is purposefully ignored so we can better identify the impact of de-contextualizing information as shown in Fig. 5.

In practice, this reduced-context model is built upon the existing CNN layers as in the E-BP case. We will create this reduced context system by employing only the E-BP embeddings. The E-BP embeddings are extracted from the same emotion system as before. In this case, however, instead of being fed to a recursive layer with full-session view, we eliminate the recursive layer and incorporate a variable number of CNN layers and local average pooling functions in between to adjust context view. Since the final max-pooling layer ignores the order of the input, the largest context is determined by the receptive field view of the last layer before this max-pooling. We can thus investigate the impact of context by varying the length of the CNN receptive field.

**Figure 6:**
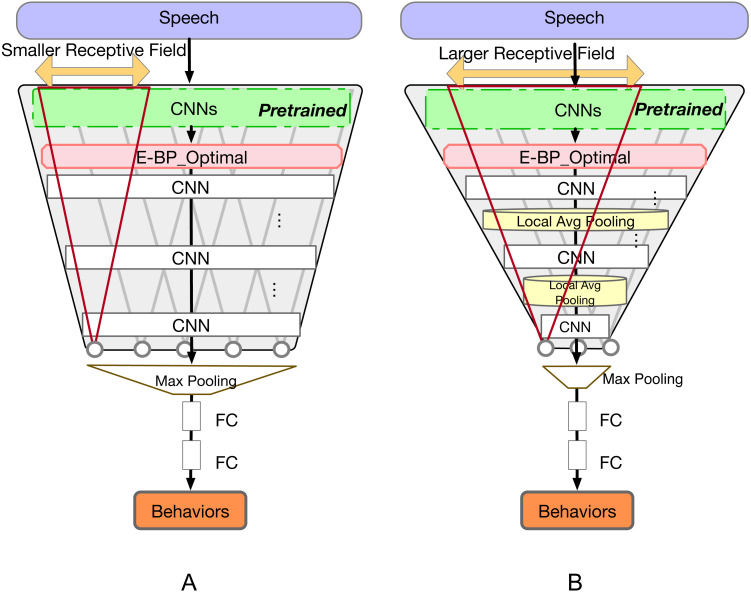
E-BP reduced context-dependent behavior recognition model. Model (A) has a smaller receptive field while model (B) has a larger receptive field because of the added local average pooling layers.

Figure 6 illustrates the model architecture. We extract the optimal E-BP based on the results of previous model, and then employ more CNN layers with different receptive field sizes to extract high-dimensional representation embeddings, and finally input them to the adaptive max-pooling along the time axis to eliminate the sequential information. Within each CNN receptive field, shown as red triangles in the figure, the model still has access to the full receptive field context. The max pooling layer removes context across the different receptive windows.

Furthermore, the receptive field can be large enough to enable the model to capture behavioral information encoded over longer timescales. In contrast a very small receptive area, e.g., at timescale of phoneme or word, sensing behaviors should be extremely difficult (Baumeister et al., 2010) and can even be challenging to detect emotions (Mower & Narayanan, 2011). The size of the receptive field is decided by the number of CNN layers, corresponding stride size, and the number of local average pooling layers in between. In our model, we adjust the size of the receptive field by setting different number of local average pooling layers under which the overall number of network parameters is unchanged.

## Datasets

### Emotion dataset: CMU-MOSEI dataset

The CMU Multimodal Opinion Sentiment and Emotion Intensity (CMU-MOSEI) (Zadeh et al., 2018) contains video files carefully chosen from YouTube. Each sample is a monologue with verified quality video and transcript. This database includes 1000 distinct speakers with 1000 kinds of topics, and are gender balanced with an average length of 7.28 seconds. Speech segments are already filtered during the data collection process, thus all speech segments are monologues of verified audio quality.

For each speech segment, six emotions (Happiness, Sadness, Anger, Fear, Disgust, Surprise) are annotated on a [0,3] Likert scale for the presence of each emotion. (0: no evidence; 1: weak evidence; 2: evidence; and 3: high evidence of emotion). This, after averaging ratings from 3 annotators, results in a 6-dimensional emotional rating vector per speech segment. CMU-MOSEI ratings can also be binarized for each emotion: if a rating is greater than 0 it is considered that there is some presence of emotion, hence it is given a true presence label, while a zero results in a false presence of the emotion.

The original dataset has 23,453 speech segments and each speech segment may contain more than one emotion presence label. Through our experiments, we use the segments with available emotion annotations and standard speaker independent split from dataset SDK (Zadeh, 2019): overall we have true presence in 12,465 segments for happiness, 5,998 for sadness, 4,997 for anger, 2,320 for surprise, 4,097 for disgust and 1,913 for fear. Due to the imbalance, accurate estimation of some emotions will be challenging. The training set consists of 16,331 speech segments, while the validation set and test set consist of 1,871 and 4,662 sentences respectively.

### Behavior dataset: couples therapy corpus

The Couples Therapy dataset is employed to evaluate complex human behaviors. The corpus was collected by researchers from the University of California, Los Angeles and the University of Washington for the Couple Therapy Research Project (Christensen et al., 2004). It includes a longitudinal study of 2 years of 134 real distressed couples. Each couple has been recorded at multiple instances over the 2 years. At the beginning of each session, a relationship-related topic (e.g., “why can’t you leave my stuff alone?”) was selected and the couple interacted about this topic for 10 minutes. Each participant’s behaviors were rated by multiple well-trained human annotators based on the Couples Interaction (Heavey, Gill & Christensen, 2002) and Social Support Interaction (Jones & Christensen, 1998) Rating Systems. 31 behavioral codes were rated on a Likert scale of 1 to 9, where 1 refers absence of the given behavior and 9 indicates a strong presence. Most of the sessions have 3 to 4 annotators, and annotator ratings were averaged to obtain the final 33-dimensional behavioral rating vector. The employed part of the dataset includes 569 coded sessions, totaling 95.8 h of data across 117 unique couples.

## Audio Processing and Feature Extraction

### Behavioral dataset pre-processing

For preprocessing the couples therapy corpus we employ the procedure described in (Black et al., 2013). The main steps are Speech Activity Detection (SAD) and diarization. Since we only focus on acoustic features extracted for speech regions, we extract the speech parts using the SAD system described in Ghosh, Tsiartas & Narayanan (2011), and only keep sessions with an average SNR greater than 5 dB (72.9% of original dataset). Since labels of behavior are provided per-speaker, accurate diarization is important in this task. Thus, for diarization we employ the manually-transcribed sessions and a forced aligner in order to achieve high quality interlocutor-to-audio alignment. This is done using the recursive ASR-based procedure of alignment of the transcripts with audio by *SailAlign* (Katsamanis et al., 2011).

Speech segments from each session for the same speaker are then used to analyze behaviors. During testing phase, a leave-test-couples-out process is employed to ensure separation of speaker, dyad, and interaction topics. More details of the preprocessing steps can be found in (Black et al., 2013).

After the processing procedure above, the resulting corpus has a total of 48.5 h of audio data across 103 unique couples and a total of 366 sessions.

### Feature extraction

In this work, we focus only on the acoustic features of speech. We utilize Log-Mel filterbank energies (Log-MFBs) and MFCCs as spectrogram features. Further, we employ pitch and energy. These have been shown in past work to be the most important features in emotion and behavior related tasks. These features are extracted using Kaldi (Povey et al., 2011) toolkit with a 25 ms analysis window and a window shift of 10 ms. The number of Mel-frequency filterbanks and MFCCS are both set to 40. For pitch, we use the extraction method in Ghahremani et al. (2014), in which 3 features, normalized cross correlation function (NCCF), pitch (*f*_0_), the delta of pitch, are included for each frame.

After feature extraction, we obtain an 84-dimensional feature per frame (40 log-MFB’s, 40 MFCC’s, energy, *f*_0_, delta of *f*_0_, and NCFF).

## Experiments and Results Discussion

### General settings

For emotion-related tasks, we utilize the CMU-MOSEI dataset with the given standard train, validation, test data split from Zadeh (2019).

For the behavior related tasks, we employ the couple therapy corpus and use leave-4-couples-out cross-validation. Note that this results in 26 distinct neural-network training-evaluation cycles for each experiment. During each fold training, we randomly split 10 couples out as a validation dataset to guide the selection of the best trained model and prevent overfitting. All these settings ensure that the behavior model is speaker independent and will not be biased by speaker characteristics or recording and channel conditions.

In our experiments, we employ five behavioral codes: *Acceptance, Blame, Positivity, Negativity* and *Sadness*, each describing a single interlocutor in each interaction of the couples therapy corpus. Table 1 lists a brief description^1^ 1Full definitions are too long to insert in this manuscript and reader is encouraged to look into (Heavey, Gill & Christensen, 2002; Jones & Christensen, 1998)of these behaviors from the annotation manuals (Heavey, Gill & Christensen, 2002; Jones & Christensen, 1998).

**Table 1:** Description of behaviors.

Behavior	Description
Acceptance	Indicates understanding, acceptance, respect for partner’s views, feelings and behaviors
Blame	Blames, accuses, criticizes partner and uses critical sarcasm and character assassinations
Positivity	Overtly expresses warmth, support, acceptance, affection, positive negotiation
Negativity	Overtly expresses rejection, defensiveness, blaming, and anger
Sadness	Cries, sighs, speaks in a soft or low tone, expresses unhappiness and disappointment

Following the same setting of (Black et al., 2010) to reduce effects of interannotator disagreement, we model the task as a binary classification task of low- and high- presence of each behavior. This also enables balancing for each behavior resulting in equal-sized classes. This is especially useful as some of the classes, e.g., Sadness, have an extremely skewed distribution towards low ratings. More information on the distribution of the data and impact on classification can be found in (Georgiou et al., 2011). Thus, for each behavior code and each gender, we filter out 70 sessions on one extreme of the code (e.g., high blame) and 70 sessions at the other extreme (e.g., low blame).

Since due to the data cleaning process, some sessions may be missing some of the behavior codes, we use a mask and train only for the available behaviors. Moreover, the models are trained to predict the binary behavior labels for all behaviors together. The loss is calculated by averaging 5 behavioral classification loss with masked labels. Thus, this loss is not optimizing for any specific behavior but it is focusing on the general, latent, link between emotions and behaviors.

### ER and EC for emotion recognition

Both the ***Multi-Emotion Regression Network (ER)*** and the ***Single-Emotion Classification Network (EC)*** are trained using the CMU-MOSEI dataset.

The ***Multi-Emotion Regression Network (ER)*** system consists of 4 layers of 1D CNN layers, adaptive max-pooling layer and followed by 3 fully connected layers with ReLU activation function. During the training, we randomly choose a segment from each utterance and represent the label of the segment using the utterance label. In our work, we employ a segment length of 1 s.

The model is trained jointly with all six emotions by optimizing the mean square error (MSE) regression loss for all emotions ratings together using Adam optimizer (Kingma & Ba, 2014).

In a stand-alone emotion regression task, a separate network that can optimize per-emotion may be needed (through higher-level disconnected network branches), however in our work, as hypothesized above, this is not necessary. Our goal is to extract as much information as possible from the signal relating to any and all available emotions. We will, however, investigate optimizing per emotion in the EC case.

Further to the ER system, we can optimize per emotion through the ***Single-Emotion Classification Network (EC)***. This is trained for each emotion separately by replacing the pre-trained ER ’s last linear layer with three emotion-specific fully connected layers. We use the same binary labeling setting as described in Zadeh et al. (2018): within each emotion, for samples with original rating value larger than zero, we assign the label 1 by considering the presence of that emotion; for samples with rating 0, we assign label 0. During training, we randomly choose 1-second segments as before. During evaluation, we segment each utterance into one-second segments and the final utterance emotion label is obtained via majority voting. In addition, the CMU-MOSEI dataset has a significant data imbalance issue: the true label in each emotion is highly under-represented. To alleviate this, during training, we balance the two classes by subsampling the 0 label esence class in every batch.

In our experiments, in order to correctly classify most of the relevant samples, the model is optimized and selected based on average weighted accuracy (WA) as used in Zadeh et al. (2018). WA is defined in Tong et al. (2017): Weighted Accuracy = (*TP* × *N*∕*P* + *TN*)∕2*N*, where *TP* (resp. *TN*) is true positive (resp. true negative) predictions, and *P* (resp. *N*) is the total number of positive (resp. negative) examples. As shown in Table 2, we present WA of each EC system and compare them with the state-of-art results from Zadeh et al. (2018).

**Table 2:** Weighted classification accuracy (WA) in percentage for emotion recognition on the CMU-MOSEI dataset. Bold numbers represent the best performing system.

Emotions	Anger	Disgust	Fear	Happy	Sad	Surprise
Methods in CMU-MOSEI						
Zadeh et al. (2018)	56.4	60.9	**62.7**	61.5	62.0	54.3
Proposed EC	**61.2**	**64.9**	57.0	**63.1**	**62.5**	**56.2**

Compared with Zadeh et al. (2018), our proposed 1D CNN based emotion recognition system achieves comparable results and thus the predicted binary emotion labels can be considered satisfactory for further experiments. More importantly, our results indicate that the pre-trained ER embedding captures sufficient emotion related information and can thus be employed as a behavior primitive.

### Context-dependent behavior recognition

The main purpose of the experiments in this subsection is to verify the relationship between emotion-related primitives and behavioral constructs. We employ both B-BP and E-BP as described below. Before that, we first use examples to illustrate the importance of context information in behavior understanding.

#### Importance of context information in behavior understanding

**Figure 7:**
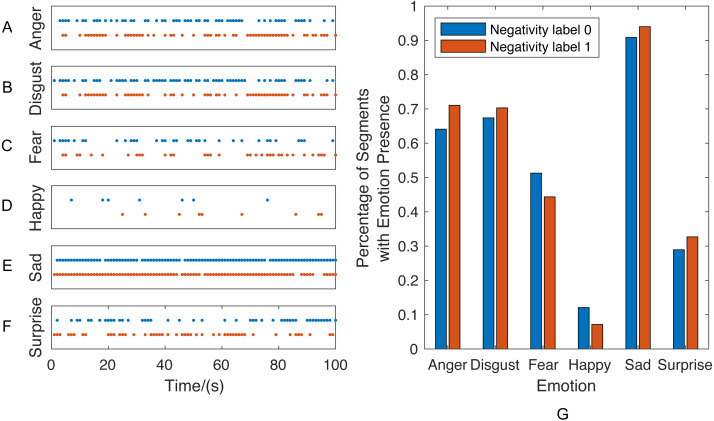
Sessions with similar percentage of emotions presence but different behavior label.

Prior to presenting the behavior classification results, we use two sessions from couple therapy corpus to illustrate the importance of context information in behavior understanding. Once the ***Single-Emotion Classification Network (EC)*** systems are trained, a sequence of emotion label vectors can be generated by applying the EC systems on each speech session. We choose two sessions and plot those sequences of emotion presence vectors of the first 100 seconds as an example in Fig. 7, in which each dot represents the emotion presence (i.e., predicted label equals to 1) at the corresponding time. For each emotion, the percentage of emotion presence segments is calculated by dividing the number of emotion presence segments by the total number of segments.

These two sessions are selected as an example since they have similar audio stream length and percentage of emotion presence segments but different behavior labels: the red represents one session with “strong presence of negativity” while blue represents another session with “absence of negativity”. This example reveals the fact that, as we expected, the behaviors are determined not only by the percentage of affective constructs but also the contextual information. As shown in the left (Figs. 7A–7F), the emotion presence vectors exhibit different sequential patterns within two sessions, even though no significant distribution difference can be observed in Fig. 7G.

#### B-BP based context-dependent behavior recognition

***Binarized*** *Emotion-Vector* ***Behavior Primitives*** are generated by applying the ***Single-Emotion Classification Network (EC)*** systems on the couple therapy data: For each session, a sequence of emotion label vectors is generated as *E* = [**e**_1_, **e**_2_, ..., **e**_***T***_], where each element **e**_*i*_ is the 6 dimensional B-BP binary label vector at time *i*. That means that *e*_*ij*_ represents the presence, through a binary label 0 or 1, of emotion *j* at time *i*. Such B-BP are the input of the context-dependent behavior recognition model that has two layers of GRUs followed by two linear layers as illustrated in Fig. 3.

As shown in Table 3, the average binary classification accuracy of these five behaviors is 60.43%. Considering that the classification accuracy can reach up to 50% by chance with balanced data, our results show that behavioral states can be weakly inferred from the emotion label vector sequences. Further, we perform the McNemar test, and the results above and throughout the paper are statistically significant with *p* < 0.01. Despite the low accuracy of the behavior positivity, these results suggest a relationship between emotions and behaviors that we investigate further below.

**Table 3:** Behavior binary classification accuracy in percentage for context-dependent behavior recognition model from emotion labels.

**Average**	Acceptance	Blame	Positivity	Negativity	Sadness
60.43	61.07	63.21	59.64	59.29	58.93

#### E-BP based context-dependent behavior recognition

The simple binary emotion vectors (as B-BP) indeed link emotions and behaviors. However, they also demonstrate that the binarized form of B-BP limits the provided information bandwidth to higher layers in the network, and as such limits the ability to predict the much more complex behaviors. These are reflected in the low accuracies in Table 3 .

This further motivates the use of the *Emotion*-Embedding Behavior Primitives. As described in Fig. 4, we construct input of the E-BP context-dependent behavior recognition system using the pretrained ***Multi-Emotion Regression Network (ER)***. These E-BP embeddings capture more information than just the binary emotion labels. They potentially capture a higher abstraction of emotional content, richer paralinguistic information, conveyed through a non-binarized version that doesn’t limit the information bandwidth, and may further capture other information such as speaker characteristics or even channel information.

We employ embeddings from different layers of the ER network. The layers before the employed embedding are in each case frozen and only the subsequent layers are trained as denoted in Fig. 4. The trainable part of the network includes several CNN layers with max pooling and subsequent GRU networks. The GRU part of the network is identical to the ones used by the context-dependent behavior recognition from E-BP.

The use of different depth embeddings can help identify where information loss becomes too specific to the ER loss objective versus where there is too much unrelated information to the behavior task.

In Table 4, the none-E-BP model, as the baseline, means all parameters are trained from random initialization instead of using the pretrained E-BP input. While E-BP_ *l* model means the first *l* layers of the pretrained ER network are fixed and their output is used as the embedding E-BP for the subsequent system. As seen in the second column of the table, all of E-BP based models perform significantly better than the B-BP based model, which achieves an improvement of 8.57% on average and up to 16.78% for Negativity.

**Table 4:** Behavior binary classification accuracy in percentage for context-dependent behavior recognition model from emotion-embeddings. Bold numbers represent the best performing system.

	**Average**	Acceptance	Blame	Positivity	Negativity	Sadness
None-E-BP model (Baseline)	58.86	62.86	62.50	57.86	60.00	51.07
E-BP_1 model	59.79	64.29	62.86	60.00	61.07	50.71
E-BP_2 model	60.79	61.79	63.93	62.86	63.57	51.79
E-BP_3 model	65.00	66.07	69.29	**65.36**	69.29	55.00
E-BP_4 model	**69.00**	**72.50**	**71.79**	**65.36**	**76.07**	**59.29**

These results, further support the use of basic emotions as constructs of behavior. In general, for all behaviors, the higher-level E-BP s, which are closer to the ER loss function, can capture affective information and obtain better performance in behavior quantification compared with lower-level embeddings. From the description in Table 1, some behaviors are closely related to emotions. For example, negativity is defined in part as “Overtly expresses rejection, defensiveness, blaming, and anger”, and sadness^2^ 2Which isn’t necessarily perfectly aligning with the basic emotion “sad” but follows the SSIRS manual.is defined in part as “expresses unhappiness and disappointment”. This shows that these behaviors are very related to emotions such as anger and sad, thus it’s expected that an embedding closer to the ER loss function will behave better. Note that these are not at all the same though: a negative behavior may mean that somewhere within the 10 min interaction or through unlocalized gestalt information the expert annotators perceived negativity; in contrast a negative emotion has short-term information (on average 7s segment) that is negative.

An interesting experiment is what happens if we use a lower-ratio of emotion (out-of-domain) vs. behavior (couples-in-domain) data. To perform this experiment we use only half of the CMU-MOSEI data^3^ 311,875 samples from commit: https://github.com/A2Zadeh/CMU-MultimodalSDK/commit/f0159144f528380898df8093381c8d83fd7cc475.to train another ER system, and use this less robust ER system and corresponding E-BP representations to reproduce the behavior quantification as in Table 4. What we observe is that the reduced learning taking place on emotional data requires the in-domain system to have prefer embeddings closer to the feature. Specifically Negativity performs equally well with layers 3 or 4 at 71.43%. Positivity performs best with layer 3 at 64.64%, Blame and Acceptance perform best with layer 2 at 71.07% and 72.86% respectively while Sadness performs best through layer 1 at 56.07%.

In the reduced data case we observe that best performing layer is not consistently layer 4. Employing the full dataset as in Table 4 provides better performance than using less data and in that case layer 4 (E-BP_4) is always the best performing layer, thus showing that more emotion data provides better ability of transfer learning.

### Reduced context-dependent behavior recognition

In the previous two sections we demonstrate that there is a benefit to transfer emotion-related knowledge to behavior tasks. We show that the wider bandwidth information transfer through an embedding E-BP is beneficial to a binarized B-BP representation. We also show that depending on the degree of relationship of the desired behavior to the signal or to the basic emotion, different layers that are closer to the input signal or closer to the output loss, may be more or less appropriate. However, in all the above cases we assume that the sequence and contextualization of the extracted emotion information was needed. That is captured and encoded through the recursive GRU layers.

We conduct an alternative investigation into how much contextual information is needed. As discussed in section Reduced context-dependent behavior recognition from emotion-informed embeddings and shown on Fig. 6 we can reduce context through changing the receptive field of our network prior to removing sequential information via max pooling.

In this section we select the best E-BP based on average results in Table 4, i.e., E-BP-4, as the input of the reduced context-dependent behavior recognition model. Based on E-BP-4 embeddings, the reduced context-dependent model employs 4 more CNN layers with optional local average pooling layers in between, and is followed by an adaptive max pooling layer and three fully connected layers to predict the session level label directly without sequential modules.

Since the number of parameters of this model is largely increased, dropout (Srivastava et al., 2014) layers are also utilized to prevent overfitting. Local average pooling layers with kernel size 2 and stride 2 are optionally added between newly added CNN layers to adjust the final size of the receptive field: the more average pooling layers we use, the larger temporal receptive field can be obtained for the same number of network parameters. We endure that the overall number of trainable parameters is the same for the different receptive field settings, which provides a fair comparison of the resulting systems. The output of these CNN/local pooling layers is passed to an adaptive max pooling before the fully connected layers as in Fig. 6.

In Table 5, each model has a different temporal receptive window ranging from 4 seconds to 1 min. For most behaviors, we observe a better classification as the receptive field size increases, especially in the range from 4 seconds to 32 s, demonstrating a need for longer observations for behaviors.

**Table 5:** Behavior binary classification accuracy in percentage for reduced context-dependent behavior recognition from emotion-informed embeddings. Bold numbers represent the best performing system.

	**Average**	Acceptance	Blame	Positivity	Negativity	Sadness
Receptive_field_ 4s	63.43	65.00	70.00	58.92	67.50	55.71
Receptive_field_ 8s	62.71	65.00	69.64	56.79	66.07	56.07
Receptive_field_ 16s	63.36	63.57	69.64	60.71	66.42	**56.43**
Receptive_field_ 32s	**66.36**	**68.21**	**73.21**	**63.21**	71.43	55.71
Receptive_field_ 64s	65.57	66.43	72.86	62.50	**71.79**	54.29

Furthermore, the results suggest different behaviors require different observation window length to be quantified, which is also observed by Chakravarthula et al. (2019) using lexical analysis. By comparing results with different receptive window sizes, we can indirectly obtain the appropriate behavior analysis window size for each behavior code. As shown in Table 5, sadness has a smaller optimal receptive field size than behaviors such as acceptance, positivity and blame. This is in good agreement with the behavior descriptions. For example, behaviors of acceptance, positivity and blame often require relatively longer observations since they relate to understanding and respect for partner’s views, positive negotiation, and accusation respectively, which often require multiple turns in a dialog and context to be captured. On the other hand, sadness which can be expressed via emitting a long, deep, audible breath, and is also related to short-term expression of unhappy affect, can be captured with shorter windows.

Moreover, we find the classification of negativity reaches high accuracy when using a large receptive field. This might be contributed by the fact that the negative behavior in the couple therapy domain is complex, which is not only revealed by short term negative affect but also related to context based negotiation and hostility, and is captured through gestalt perception of the interaction.

In addition, the conclusion that most of the behaviors do not benefit much from longer than 30 s^4^ 4Note that this does not make any claims on interlocutor dynamics, talk time, turn-taking etc., but just single person acoustics.windows matched existing literature on thin slices (Ambady & Rosenthal, 1992), which refer to excerpts of an interaction that can be used to arrive at a similar judgment of behavior to as if the entire interaction had been used.

### Analysis on behavior prediction uncertainty reduction

Besides the verification of the improvement from B-BP based model to E-BP based models, in this section, we further analyze the importance of context information for each behavior by comparing results between E-BP based context-dependent and reduced context-dependent models. This analysis calls into question that which behavior is more context involved and to what degree.

Classification accuracy is used as the evaluation criterion in previous experiments. More generally, this number can be regarded as a probability of correct classification when a new session comes to measure. Inspired by entropy from information theory, we define one metric named Prediction Uncertainty Reduction (PUR) and use it to indicate the relative behavior prediction and interpretation improvement among different models for each behavior.

Suppose *p*_*m*_(*x*) ∈ [0, 1] is the probability of correct classification for behavior *x* with model *m*. We define the uncertainty of behavior prediction as:

\begin{eqnarray*}{I}_{m}(x)=-{p}_{m}(x)lo{g}_{2}({p}_{m}(x))-(1-{p}_{m}(x))lo{g}_{2}(1-{p}_{m}(x)) \end{eqnarray*}

if *p*_*m*_(*x*) is equal to 1, *I*_*m*_(*x*) = 0 there is no improvement possibility; if *p*_*m*_(*x*) is equal to 0.5, same as random prediction accuracy, the uncertainty is the largest. We further define the Prediction Uncertainty Reduction (PUR) value of behavior *x* from model *m* to model *n* as:

\begin{eqnarray*}{R}_{m\rightarrow n}(x)={I}_{m}(x)-{I}_{n}(x). \end{eqnarray*}

We use this value to indicate improvements between different models.

We use PUR to sense the relative improvement from E-BP based context-dependent and E-BP based reduced context-dependent models respectively, to the baseline B-BP based context-dependent model. The larger value of PUR suggests the clear improvement of behavior prediction. For each behavior, for each E-BP based model, we choose the best performance model (the bold number from Tables 4 and 5) to calculate PUR value from baseline B-BP context-dependent model.

**Figure 8:**
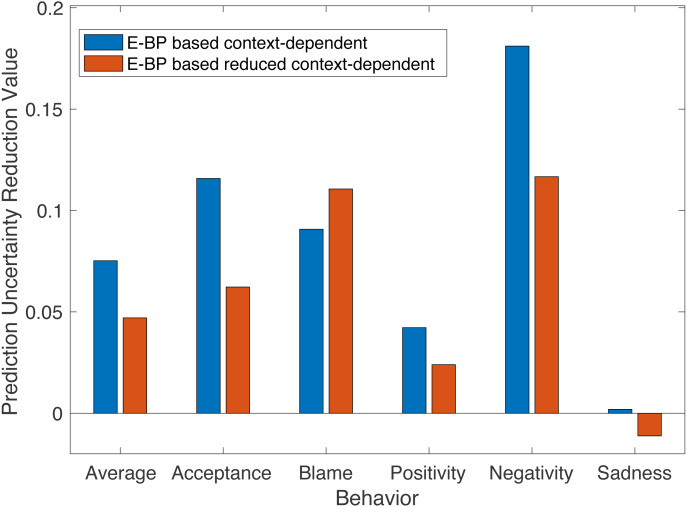
PUR optimal value of E-BP based context-dependent and reduced context-dependent models across behaviors.

In Fig. 8, as expected, for most behaviors the positive PUR values verify the improvement from using informative E-BP to simple binary B-BP. In addition, the results support the hypothesis that the sequential order of affective states is one non-negligible factor of behavior analysis since the PRU of context-dependent (blue color) model is better than that of reduced context one (red color) for most behaviors.

More interestingly, for each behavior, the difference between two bars (i.e., PUR difference) can imply the necessity and importance of the sequential and contextual factor of quantifying that behavior. We notice that for “positive” or more “complex problem solving” related behaviors (e.g., Acceptance, Positivity), the context based model can achieve better performance than the reduced context model. While the PUR differences from “negative” related behaviors (e.g., Blame, Negativity) varies from different behaviors. For example, the behavior of acceptance, with a large PUR difference, it is more related to “understanding, respect for partner’s views, feelings and behaviors”, which could involve more turns in a dialog and context information. In addition, positivity requires the monitoring of consistent positive behavior, since a single negative instance within a long positive time interval would still reduce positivity to a very low rating.

In contrast, we see that although blame can still benefit from a larger contextual window, there is no benefit to employing the full context. This may infer that blame expression is more localized.

Furthermore, our findings are also congruent with many domain annotation processes: some behaviors are potentially dominated by salient information with short range, and one short duration appearance can have a significant impact on the whole behavior rating, while some behaviors need longer context to analyze (Heavey, Gill & Christensen, 2002; Jones & Christensen, 1998).

However, among all behaviors, “sadness” is always the hardest one to predict with high accuracy, and there is little improvement after introducing different BPs. This could be resulting from the extremely skewed distribution towards low ratings as mentioned in above and (Georgiou et al., 2011; Black et al., 2013), which leads to a very blurred binary classification boundary compared to other behaviors. The detailed network architecture and training parameters are shown in the appendix from Tables A1–A5.

## Conclusion and Future Work

In this work, we explored the relationship between emotion and behavior states, and further employed emotions as behavioral primitives in behavior classification. In our designed systems, we first verified the existing connection between basic emotions and behaviors, then further verified the effectiveness of utilizing emotions as behavior primitive embeddings for behavior quantification through transfer learning. Moreover, we designed a reduced context model to investigate the importance of context information in behavior quantification.

Through our models, we additionally investigated the empirical analysis window size for speech behavior understanding, and verified the hypothesis that the order of affective states is an important factor for behavior analysis. We provided experimental evidence and systematic analyses for behavior understanding via emotion information.

To summarized, we investigated three questions and we concluded:

1. Can the basic emotion states infer behaviors? The answer is yes. Behavioral states can be weakly inferred from emotions states. However behavior requires richer information than just binary emotions.
2. Can emotion-informed embeddings be employed in the prediction of behaviors? The answer is yes. The rich emotion involved embedding representation helps the prediction of behaviors. They also do so much better than the information-bottlenecked binary emotions.
3. Is the contextual (sequential) information important in defining behaviors? The answer is yes. We verify the importance of context of behavior indicators for all behaviors. Some behaviors benefit from incorporating the full interaction (10 minutes) length while others require as little as 16 seconds of information, but all perform best when given contextual information.

Moreover, the proposed neural network systems are not limited to the datasets and domains of this work, but potentially provides a path for investigating a range of problems, such as local versus global, sequential versus non-sequential comparisons in many related areas. In addition to the relationship of emotions to behaviors, a range of other cues can also be incorporated towards behavior quantification. Moreover, many other aspects of behavior, such as entrainment, turn-taking duration, pauses, non-verbal vocalizations, and influence between interlocutors, can be incorporated. Many such additional features can be similarly developed on different data and employed as primitives; for example entrainment measures can be trained through unlabeled data (Nasir et al., 2018).

Furthermore, we expect that the results of behavior classification accuracy maybe be further improved through improved architectures, parameter tuning, and data engineering for each behavior of interest. In addition, behavior primitives, e.g., from emotions, can also be employed via the lexical and visual modalities.

## Appendix A. Detailed network architecture and training parameters

**Table A1:** Network architecture of ER.

Multi-Emotion Regression Network (ER) framework (Input: 84 * 100 ; Output:6)
Training details: Adam optimizer(lr = 1e–05), batch size 16, MSELoss
Conv1d(in_ch=84, out_ch=96, kernel size=10, stride=2, padding=0) ReLU
Conv1d(in_ch=96, out_ch=96, kernel size=5, stride=2, padding=0) ReLU
Conv1d(in_ch=96, out_ch=96, kernel size=5, stride=2, padding=0) ReLU
Conv1d(in_ch=96, out_ch=128, kernel size=3, stride=2, padding=0) ReLU
AdaptiveMaxPool1d(1)
Linear(in =128, out =128) ReLU
Linear(in =128, out =128) ReLU
Linear(in =128, out =6)

**Table A2:** Network architecture of EC.

Single-Emotion Classification Network (EC) framework (Input: 84 * 100 ; Output: 2)	
Training details: Adam optimizer(lr = 1e–05), CrossEntropyLoss, batch size: 32; 64; 128	
Conv1d(in_ch=84, out_ch=96, kernel size=10, stride=2, padding=0) ReLU	Pretrained
Conv1d(in_ch=96, out_ch=96, kernel size=5, stride=2, padding=0) ReLU	
Conv1d(in_ch=96, out_ch=96, kernel size=5, stride=2, padding=0) ReLU	
Conv1d(in_ch=96, out_ch=128, kernel size=3, stride=2, padding=0) ReLU	
AdaptiveMaxPool1d(1)	
Linear(in =128, out =128) ReLU	
Linear(in =128, out =128) ReLU	
Linear(in =128, out =64) PReLU	Trainable
Linear(in =64, out =64) PReLU	
Linear(in =64, out =2)	

**Table A3:** B-BP based context-dependent behavior recognition model framework.

E-BP based context-dependent behavior recognition model (Input: seq_len*6; Output: 5)	
Training details: Adam optimizer(lr = 1e–04) + Polynomial learning rate decay,
Masked BCEWithLogitsLoss, batch size: 1	
Emotion recognition framework	Pretrained
GRU(in_size =6, hidden_size = 128, num_layers=2)	
Linear(in =128, out =64) ReLU	Trainable
Linear(in =64, out =5)	

**Table A4:** E-BP based context-dependent behavior recognition model framework.

E-BP based context-dependent behavior recognition model (Input: seq_len * 84 * 100 ; Output: 5)
Training details: Adam optimizer(lr = 1e–05) + Polynomial learning rate decay,
Masked BCEWithLogitsLoss, batch size: 1, epochs=300	
Conv1d(in_ch=84, out_ch=96, kernel size=10, stride=2, padding=0) ReLU	Partly pretrained Partly trainable
Conv1d(in_ch=96, out_ch=96, kernel size=5, stride=2, padding=0) ReLU	
Conv1d(in_ch=96, out_ch=96, kernel size=5, stride=2, padding=0) ReLU	
Conv1d(in_ch=96, out_ch=128, kernel size=3, stride=2, padding=0) ReLU	
AdaptiveMaxPool1d(1)	
GRU(in_size =128, hidden_size = 128, num_layers=2)	Trainable
Linear(in =128, out =64) ReLU	
Linear(in =64, out =5)	

**Table A5:** E-BP based reduced context-dependent behavior recognition model framework. Those AvgPool1d layers are optional to adjust temporal receptive field size.

E-BP based reduced context-dependent behavior recognition model (Input: 84 * seq_len ; Output: 5)
Training details: Adam optimizer(lr = 1 e–04) + Polynomial learning rate decay,	
Masked BCEWithLogitsLoss, batch size: 48, epochs=350	
Conv1d(in_ch=84, out_ch=96, kernel size=10, stride=2, padding=0) ReLU	Behavior primitive embedding (Pretrained)
Conv1d(in_ch=96, out_ch=96, kernel size=5, stride=2, padding=0) ReLU	
Conv1d(in_ch=96, out_ch=96, kernel size=5, stride=2, padding=0) ReLU	
Conv1d(in_ch=96, out_ch=128, kernel size=3, stride=2, padding=0) ReLU	
Conv1d(in_ch=128, out_ch=96, kernel size=3, stride=2, padding=0)	Trainable
**AvgPool1d(kernel size=2, stride=2)** ReLU	
Dropout(prob=0.4)	
Conv1d(in_ch=96, out_ch=96, kernel size=3, stride=2, padding=0)	
**AvgPool1d(kernel size=2, stride=2)** ReLU	
Dropout(prob=0.4)	
Conv1d(in_ch=96, out_ch=96, kernel size=3, stride=1, padding=0)	
**AvgPool1d(kernel size=2, stride=2)** ReLU	
Dropout(prob=0.4)	
Conv1d(in_ch=96, out_ch=128, kernel size=3, stride=1, padding=0)	
**AvgPool1d(kernel size=2, stride=2)** ReLU	
Dropout(prob=0.5)	
AdaptiveMaxPool1d(1)	
Linear(in =128, out =128) ReLU	
Linear(in =128, out =64) ReLU	
Linear(in =64, out =5)	

## Additional Information and Declarations

### Data Availability

The following information was supplied regarding data availability: The code used in this work is available at https://bitbucket.org/georgiou/emotions_as_primitives_towards_behavior_understanding. The Couples Therapy Corpus involves human subjects participating in real couple therapy interactions and as such is protected under an Institutional Review Board (IRB). Information on obtaining IRB clearance and access to the corpus can be obtained by contacting the author: Haoqi Li, haoqili@usc.edu.
